# Supplementary material for: Regulation of miR-181a expression in T cell aging
Source: Nat Commun. 2018 Aug 3;9:3060. doi: 10.1038/s41467-018-05552-3 (PMC6076328; doi:10.1038/s41467-018-05552-3)
Supplement: Supplementary file 1 — Supplementary Information [file 41467_2018_5552_MOESM1_ESM.pdf]

Regulation of *miR-181a* expression in T cell aging

Ye et al.

Supplementary Information

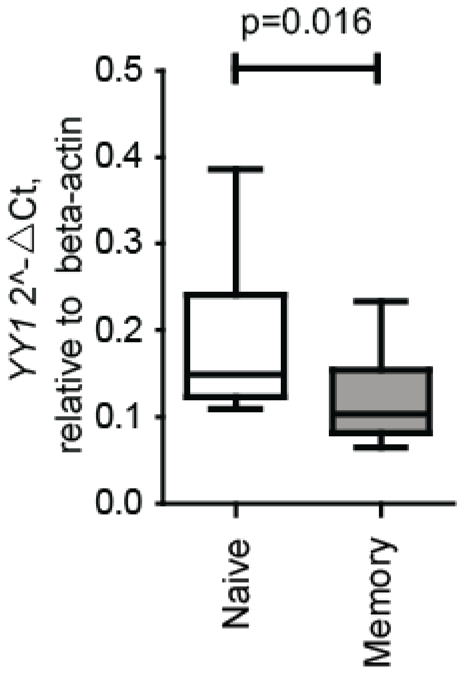

Supplementary Figure 1. *YY1* expression in naïve and memory CD4 T cells.

Naïve and memory CD4 T cells were isolated from PBMC of healthy individuals (n=14) and *YY1* transcripts were quantified by qPCR. Results are shown as box plots of *YY1* transcripts relative to β-actin. *YY1* expression was significantly higher in naïve CD4 T cells, resembling the pattern seen for miR-181a (p=0.016 by paired t-test).

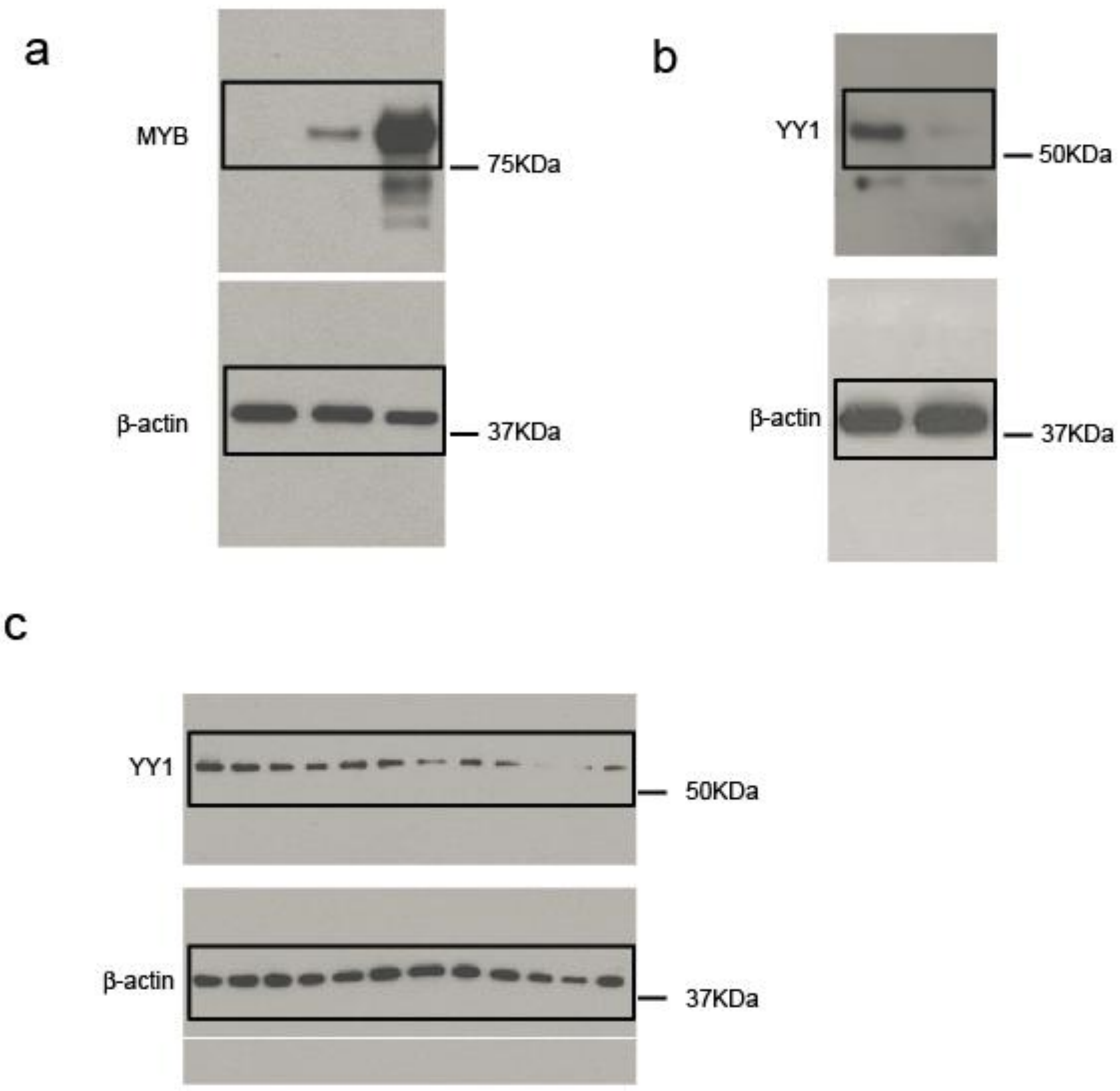

Supplementary Figure 2. Original immunoblot data of YY1 and MYB.

(a) Original immunoblots corresponding to Fig. 2b. (b) Original immunoblots corresponding to Fig. 2d. (c) Original immunoblots corresponding to Fig. 3c.

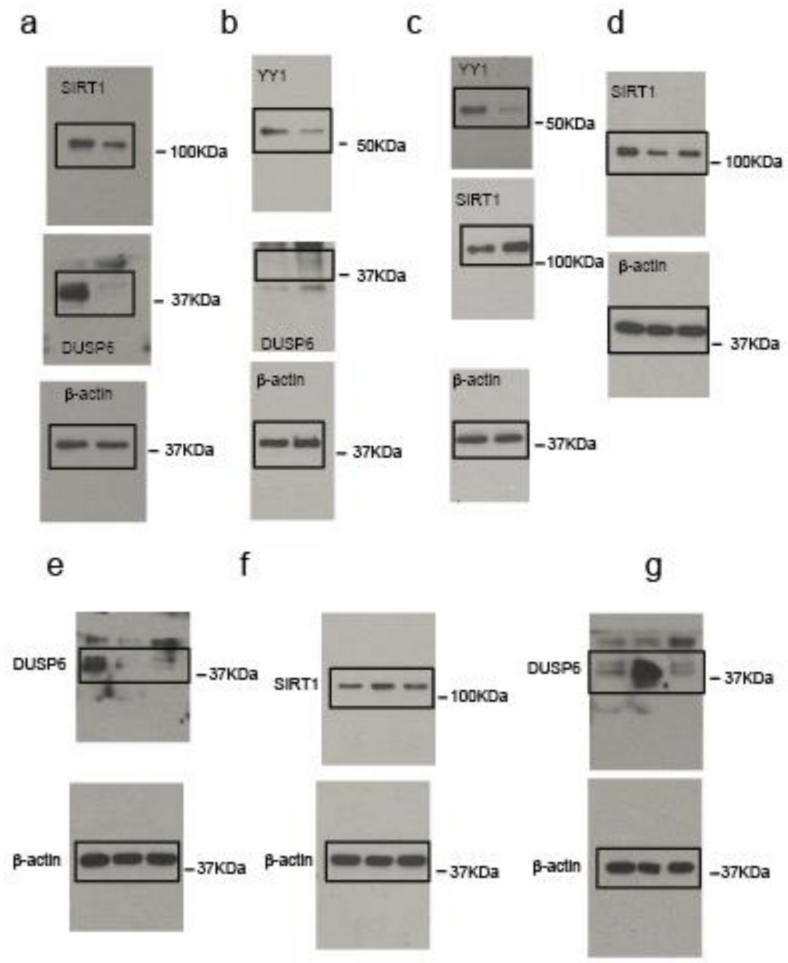

Supplementary Figure 3. Original immunoblots of YY1 and miR-181a targets.

(a) Original immunoblots corresponding to Fig. 4a. (b) Original immunoblots corresponding to Fig. 4b. (c) Original immunoblots corresponding to Fig. 4c. (d) Original immunoblots corresponding to Fig. 4d. (e) Original immunoblots corresponding to Fig. 4e. (f) Original immunoblots corresponding to Fig. 4f. (g) Original immunoblots corresponding to Fig. 4g.

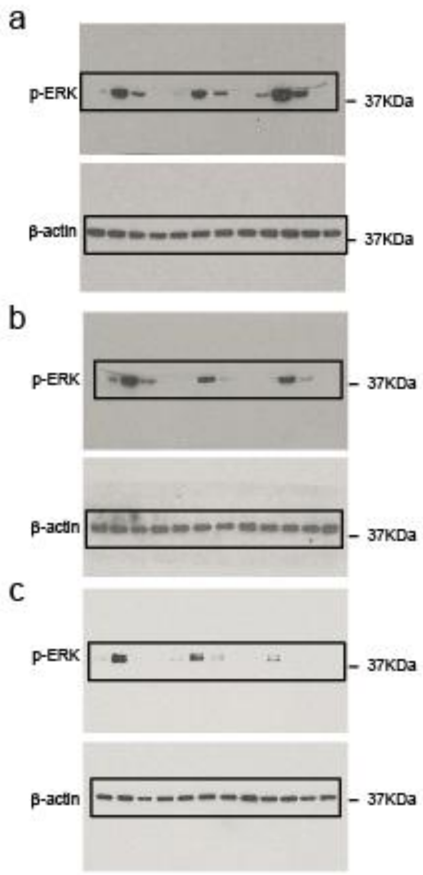

Supplementary Figure 4. Original immunoblots of phosphorylated ERK.

(a) Original immunoblots corresponding to Fig. 5a. (b) Original immunoblots corresponding to Fig .5b. (c) Original immunoblots corresponding to Fig. 5c.

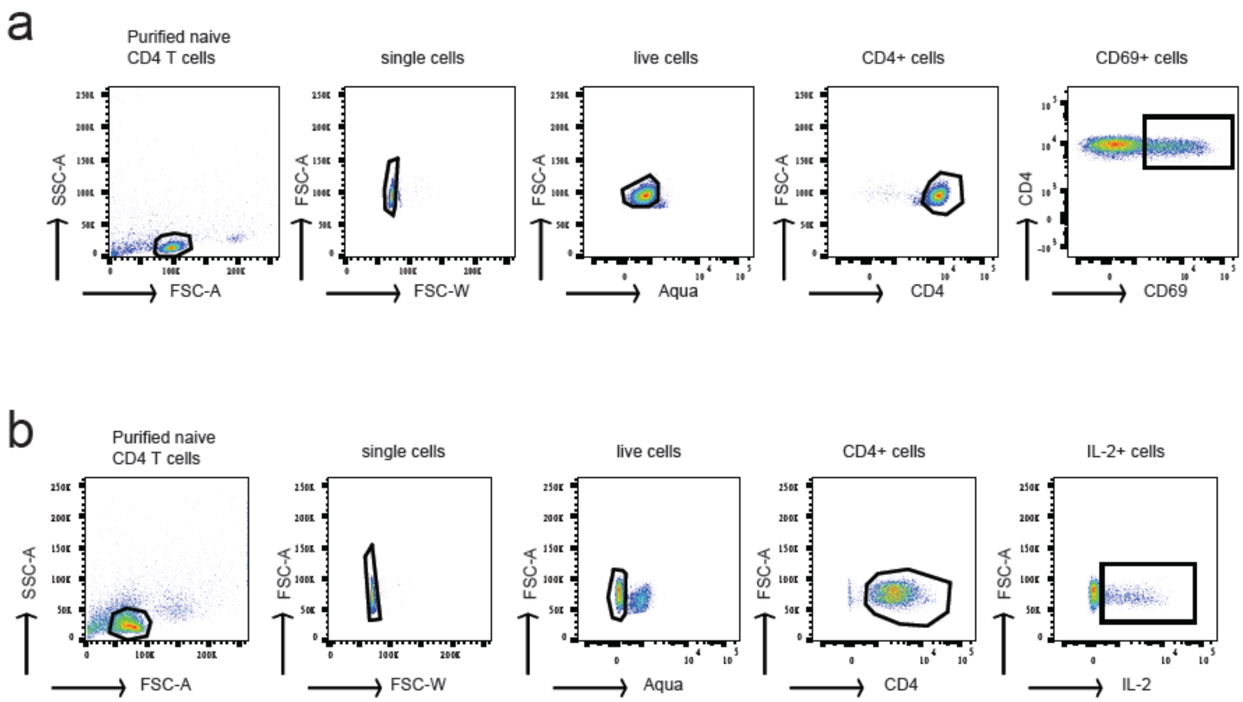

Supplementary Figure 5. Gating strategies for flow cytometric studies of CD69 expression and IL-2 production.

(a) Gating strategies for flow cytometric studies of purified naïve CD4 T cells for CD69 expression corresponding to Fig. 6a. (b) Gating strategies for flow cytometric studies of purified naïve CD4 T cells for IL2 production corresponding to Fig. 6b.

Supplementary Table 1. Differentially expressed genes in *YY1*-silenced T cells (corresponding to Fig. 3)

Gene	LogCPM	LogFC	Adjusted p-value
YY1	6.882	-1.714	2.67E-25
SESN3	10.21	0.5732	2.29E-22
RPS29	8.546	0.7576	1.66E-14
FTH1	8.73	-0.5093	1.45E-10
PRKCB	8.495	-0.4265	6.70E-07
TM9SF3	7.925	0.4054	0.000153
TXNRD1	8.356	-0.3548	0.000246
FYB	9.839	0.2356	0.000284
IQGAP2	6.819	0.505	0.000502
ITGA4	6.666	0.5311	0.000501
LEPROT	6.735	-0.5166	0.000502
RPS27	11.36	0.1453	0.000514
SERBP1	8.662	0.3108	0.000918
PTMA	9.711	-0.2241	0.0011
MYH9	10.26	-0.1941	0.001117
TTC3	8.349	0.3169	0.001117
SRRM2	10.09	-0.2024	0.001258
SLC7A5	7.146	-0.4134	0.002534
EEF1A1	13.23	0.08109	0.005202
PLEC	10.63	-0.1609	0.008262
HIPK1	9.067	-0.2328	0.009272
POLR2A	9.391	-0.2183	0.009272
RPS7	8.658	0.2647	0.009272
ZBTB44	8.278	0.2861	0.01012
RPS23	9.946	-0.1839	0.0105
EIF4G2	10.03	-0.1745	0.01108
RMRP	13.38	-0.07112	0.0152
GNA13	9.043	0.2258	0.01539
ERAP1	7.607	0.3186	0.0165
SPCS3	7.525	0.3213	0.02165
HIST1H2BK	7.01	-0.3563	0.03708
NR3C2	6.719	0.3926	0.03708
SORL1	10.48	0.1411	0.03708
RPS9	9.321	0.2048	0.04092
GSK3B	7.296	0.3216	0.04106
RABAC1	5.148	-0.6292	0.04106
GYPC	7.001	0.358	0.0424
FTL	11.38	-0.1097	0.04349
SMAD3	8.044	0.2671	0.04349
